# Aberrant STING activation promotes macrophage senescence by suppressing autophagy in vascular aging from diabetes

**DOI:** 10.1016/j.isci.2024.111594

**Published:** 2024-12-13

**Authors:** Huiqing Ding, Quan Zhang, Rukai Yang, Liyao Fu, Hejun Jiang, Qingyi Zhu, Shi Tai

**Affiliations:** 1 Department of Cardiology, The Second Xiangya Hospital of Central South University, Changsha 410011, China; 2 Department of Blood Transfusion, The Second Xiangya Hospital of Central South University, Changsha, China

**Keywords:** Molecular biology, Immunology, Cell biology

## Abstract

Diabetic vascular aging is driven by macrophage senescence, which propagates senescence-associated secretory phenotypes (SASP), exacerbating vascular dysfunction. This study utilized a type 2 diabetes mellitus (T2DM) mouse model induced by streptozotocin injection and a high-fat diet to investigate the role of STING in macrophage senescence. Vascular aging markers and senescent macrophages were assessed *in vivo*, while *in vitro*, high glucose treatment induced macrophage senescence, enhancing senescence in co-cultured vascular smooth muscle cells. Mechanistic studies revealed that STING activation inhibits autophagy by phosphorylating ULK1 at S757, accelerating senescence. Pharmacological modulation showed that the STING inhibitor H-151 alleviates, while the agonist DMXAA enhances, senescence. These findings highlight the STING-autophagy axis as a critical driver of macrophage senescence, offering insights into the molecular mechanisms of diabetic vascular aging and identifying potential therapeutic targets to mitigate vascular complications in diabetes.

## Highlights

• Vascular aging and macrophage senescence are promoted in diabetes
• STING activation promotes macrophage senescence
• STING activation inhibits autophagy through ULK1 S757 phosphorylation

## Graphical abstract

**Figure:**
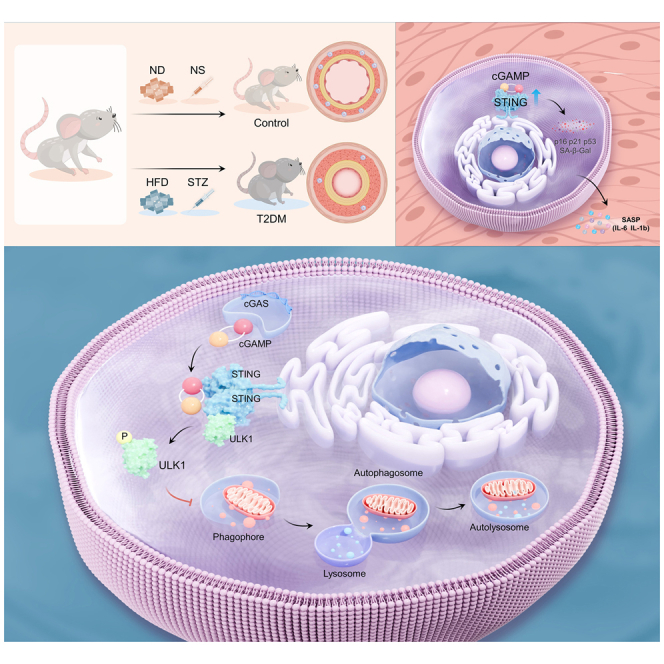

## Introduction

Age-related diseases such as cardiovascular disease, Parkinson’s disease, and osteoporosis are more prevalent in patients with T2DM,^1^^,^^2^^,^^3^ suggesting an association with premature aging.^4^ Various complications are clustered in these patients,^5^ hence, addressing a single complication does not fully enhance the quality of life or survival. Vascular aging, a common mechanism underlying many diabetes-related complications,^6^ is a significant risk factor for atherosclerosis, hypertension, stroke, and atrial fibrillation. These conditions affect the threshold, course, severity, and prognosis of cardiovascular diseases.^7^^,^^8^ Nevertheless, the impact of diabetes on vascular aging requires further investigation.

Immunometabolic studies have established a clear link between metabolic status and immune processes.^9^^,^^10^ Individuals with T2DM are more susceptible to severe, prolonged infections^11^^,^^12^ and exhibit reduced vaccine responses,^13^ indicating potential immune cell dysfunction. In addition, macrophages, key carriers of the SASP, drive chronic inflammation when senescent, significantly impacting diabetic complication progression. Research on diabetic periodontal lesions supports the hypothesis that macrophage senescence contributes to their pathogenesis.^14^ Macrophages from young diabetic mice showed extensive cell cycle inhibition and a pronounced SASP, akin to that in older mice.^14^ Early atherosclerotic lesions feature senescent foamy macrophages accumulating subendothelially, marked by increased inflammatory cytokines and chemokines, hastening atherosclerosis.^15^ In advanced lesions, senescent cells heighten plaque instability by elevating metallo-matrix protease production, which degrades elastic fibers and thins the fibrous cap.^15^ The presence of a senescent phenotype in diabetic mice’s vasculature macrophages and strategies to delay this senescence were the focus of this study.

Despite immune sensor activation in patients with diabetes by hyperglycemia and dyslipidemia via damage-associated molecular patterns, including proteins, peptides, and ions, these ligands often evade detection due to modification.^16^^,^^17^ Conversely, nucleic acids, with their stable composition and structure, are reliably recognized. Upon binding to dsDNA, cGAS is activated and produces cyclic GMP-AMP (cGAMP), which subsequently activates STING.^18^ Numerous studies have confirmed stable STING activation in diabetes.^19^^,^^20^^,^^21^ Furthermore, research indicates that the STING signaling pathway contributes to cellular senescence and SASP, whether induced by radiation, oncogene activation, or pharmacological agents.^22^^,^^23^ These findings suggest that STING pathway activation may be a common feature of stress-induced premature senescence. Among the molecular changes associated with aging, autophagy inhibition is recognized as a critical aging characteristic across different species. When stimulated by cGAMP, the anemone nematode STING induces autophagy without producing interferon, suggesting that autophagy may represent a primordial STING function.^24^ Recent studies show that STING and PtdIns3P compete for WIPI2, inhibiting each other’s autophagy pathways.^25^ Moreover, recent findings demonstrate that STING overexpression can reverse autophagy activation induced by aortic binding surgery, thus preventing cardiac remodeling and dysfunction.^26^ However, the role of STING and its regulatory mechanisms in macrophage senescence and autophagy remains largely unexplored.

In this study, we investigated the role of senescent macrophages in diabetic vascular aging and the underlying mechanisms of macrophage senescence. Our findings reveal the regulatory function of STING activation in macrophage senescence, with autophagy inhibition playing a significant role. Elucidating the pathways by which senescent macrophages contribute to diabetic vascular aging and the mechanisms of macrophage senescence will aid in developing effective therapeutic strategies to mitigate vascular lesions in patients with T2DM.

## Results

### Accelerated vascular aging in type 2 diabetes mellitus

**Figure 1:**
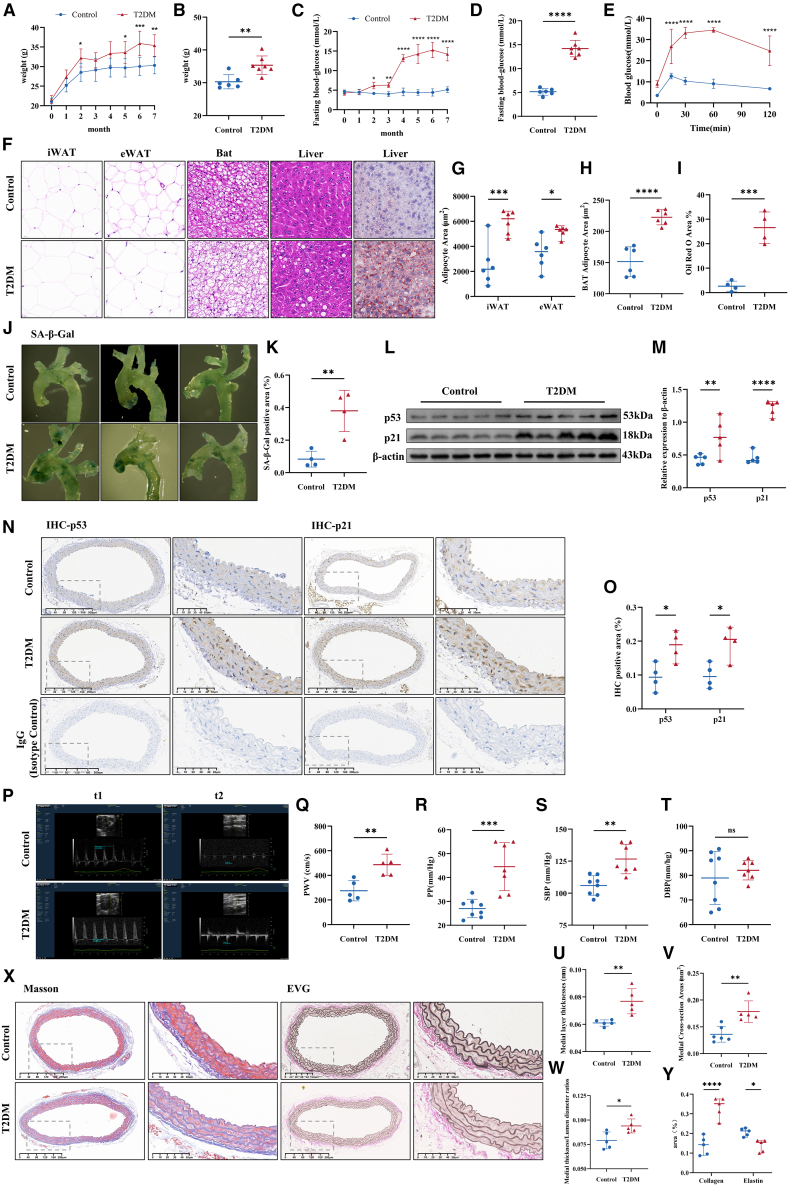
Accelerated vascular aging in T2DM (A and B) Body weight of Control (*n* = 6) and T2DM (*n* = 7) mice. (C and D) Fasting blood glucose levels in control (*n* = 6) and T2DM (*n* = 7) mice. (E)IPGTT demonstrates impaired glucose handling in T2DM mice. (F) HE staining of iWAT, eWAT, BAT, and liver, along with Oil Red O staining of liver. (G and H) Quantification of adipocyte area in iWAT and eWAT (*n* = 6). (I) Quantitative analysis of the positive area of Oil Red O staining in the liver (*n* = 4). (J and K) Representative SA-β-Gal staining images of vasculature from Control and T2DM mice (*n* = 4). (L and M) Representative western blot and analysis of p53 and p21 in vascular tissues. (*n* = 6). (N and O) Immunohistochemistry for p53 and p21 in vascular tissues. (*n* = 4, Scale bars: 200μm or 50μm). (P and Q) PWV measurement, a reliable non-invasive method, provides significant insights into vascular aging (*n* = 5). (R–T) PP, SBP, and DBP were determined using non-invasive techniques (*n* = 6). (U–W) Medial layer thicknesses, cross-sectional areas, and thickness/lumen diameter ratios were calculated (n = 5–6). (X and Y) Collagen and elastic fibers were detected using Masson and van Gieson staining, respectively (*n* = 5). Data are presented as mean ± SD. ∗*p* < 0.05; ∗∗*p* < 0.01; ∗∗∗*p* < 0.001; ∗∗∗∗*p* < 0.0001.

To evaluate vascular aging in T2DM, a comparative study was conducted between healthy mice and T2DM mice nourished on an HFD with intraperitoneal STZ administration. T2DM mice displayed higher body weight and fasting blood glucose (Figures 1A–1D). Post-STZ injection, they exhibited glucose tolerance deficits, characterized by steep glucose increases and slow decreases during the glucose tolerance test (Figure 1E). Type 2 diabetes is commonly associated with dysregulated lipid metabolism^27^^,^^28^; therefore, we conducted a detailed assessment of lipid metabolism in mice. We performed HE staining on inguinal white adipose tissue (iWAT), epididymal white adipose tissue (eWAT), brown adipose tissue (BAT), and liver samples, as well as Oil Red O staining for the liver, to assess lipid metabolism in diabetic mice (Figure 1F). The results demonstrated a significant increase in adipocyte area in both iWAT and eWAT, whereas the adipose area in BAT was notably reduced (Figures 1G and 1H). Additionally, Oil Red O staining results indicated a substantial lipid accumulation in the livers of diabetic mice (Figure 1I). Total cholesterol detection revealed markedly elevated cholesterol levels in the livers of diabetic mice (Figure S1A), further supporting the observation of disrupted lipid metabolism.

To validate vascular aging, a comprehensive analysis involving aging markers, vascular function, and structural integrity was imperative.^29^^,^^30^ SA-β-Gal staining and quantitative analysis of p53 and p21 expression levels were performed. A significant increase in the SA-β-Gal positive area was observed in the aortic arch of T2DM mice compared to healthy counterparts (Figures 1J and 1K). Elevated levels of p53 and p21 in the aorta of T2DM mice were revealed by Western blots (Figures 1L and 1M), and confirmed by immunohistochemical staining (Figures 1N and 1O). Measurements of PWV and blood pressure were conducted to evaluate the impact of diabetes mellitus on vascular function. PWV, a valid tool for the non-invasive quantification of arterial stiffness, was higher in T2DM mice compared to age-matched control mice (Figures 1P and 1Q). Notably, except for diastolic blood pressure (DBP), diabetic mice showed significantly elevated levels of differential pulse pressure (PP) and systolic blood pressure (SBP) compared to healthy counterparts (Figures 1R–1T).

**Figure 2:**
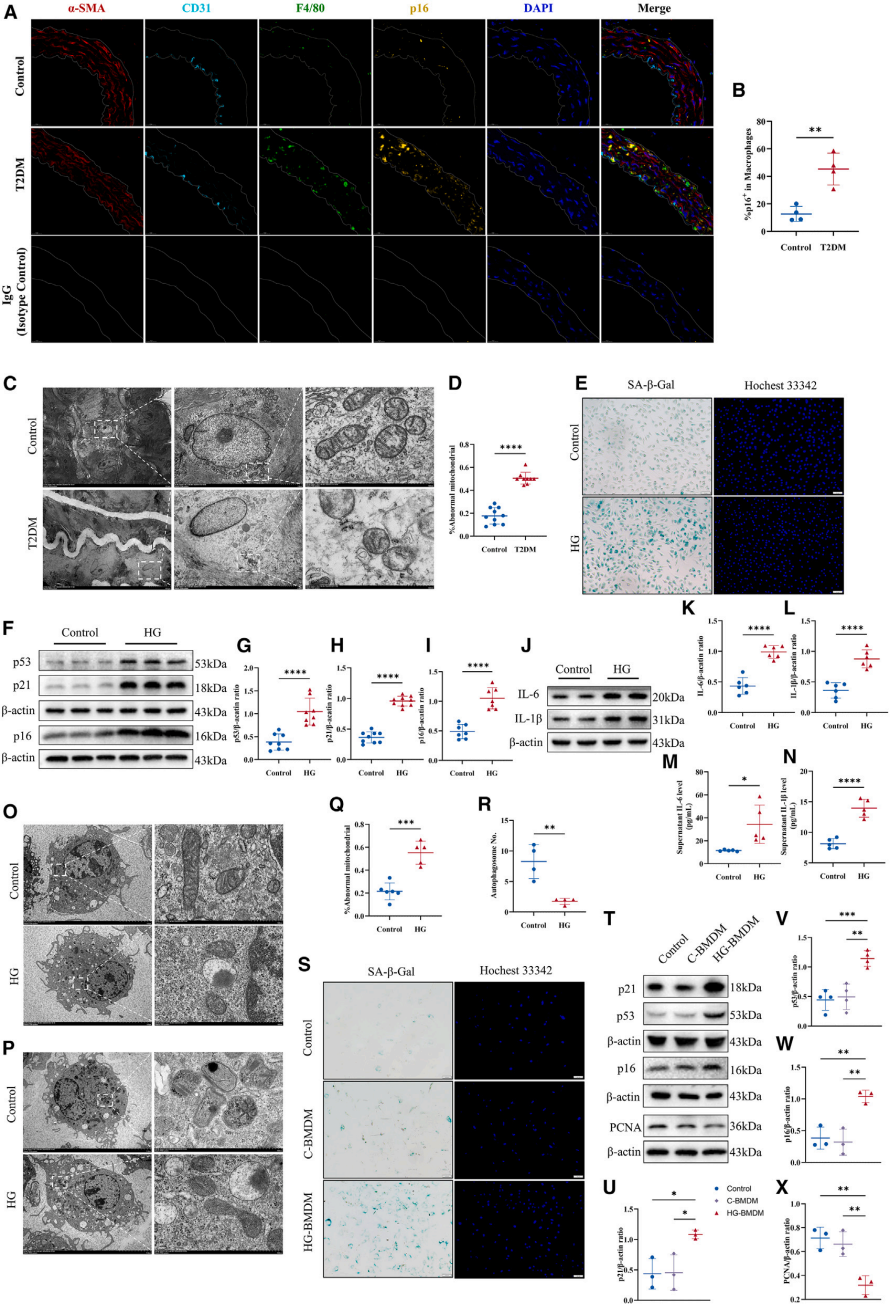
Senescent macrophages accelerate VSMCs senescence (A) IF staining of aortic samples from diabetic and healthy mice was conducted to examine senescent macrophages and vascular aging in T2DM. Markers used included α-SMA, CD31, F4/80, and p16. Scale bar: 20μm. (B) Comparative analysis of p16 expression levels in macrophages in the aortas of the control and diabetic groups, using the results from the negative control antibody as a baseline (*n* = 4). (C and D) Representative images of mitochondrial morphology. Data are displayed as a dot plot of the percentage of damaged mitochondria (n = 9–10; scale bar: 10μm, 2μm or 500nm). (E) SA-β-gal staining identifies senescent BMDMs, with nuclei stained using Hochest33342. Scale bar: 50μm. (F–L) Protein levels of p53, p21, p16, IL-6, and IL-1β were assessed using immunoblotting, with β-actin as a loading control. (n = 6–9). (M and N) ELISA analysis of IL-6 and IL-1β levels in the supernatant of cultured macrophage media. (O–R) Representative images of mitochondrial and autophagosome morphology. Data are displayed as a dot plot of the percentage of damaged mitochondria and the number of autophagosomes (n = 4–6; scale bar: 5μm or 500nm). (S) SA-β-gal staining identifies senescent vascular smooth muscle cells, with nuclei stained using Hochest33342. Scale bar: 50μm. (T–X) A co-culture model of macrophages and vascular smooth muscle cells was established. p53，p21，p16, and PCNA protein levels were assessed by immunoblotting, with β-actin as a loading control. (*n* = 3). Control, Vascular smooth muscle cells cultured separately; C-BMDM, Co-culture of vascular smooth muscle cells with control BMDM; HG-BMDM, Co-culture of vascular smooth muscle cells with HG intervention BMDM. Data are presented as means ± SD. ∗*p* < 0.05; ∗∗*p* < 0.01; ∗∗∗*p* < 0.001; ∗∗∗∗*p* < 0.0001.

Vascular aging manifested in structural alterations, including arterial wall thickening, collagen accumulation, and elastin fiber degradation, all of which resulted in decreased vessel flexibility and increased stiffness.^31^^,^^32^ HE, Masson trichrome, and Elastica Van Gieson staining techniques were employed to visualize aortic tissue rings. Vascular morphology was examined using HE and Masson’s trichrome stains in both T2DM and healthy mice cohorts. The aorta in T2DM mice demonstrated a significant increase in medial thickness and cross-sectional area compared to age-matched healthy controls (Figures 1U and 1V). An elevated ratio of media thickness to lumen diameter, commonly known as the vessel wall-to-lumen ratio, indicates abnormal vascular anatomy associated with conditions such as atherosclerosis and hypertension.^33^^,^^34^ Analysis showed a significant increase in this ratio in diabetic mice (Figure 2W). Further, Masson trichrome and Elastica Van Gieson staining analyses revealed increased vascular collagen fiber content and reduced elastic fiber thickness in T2DM mice (Figures 1X and 1Y).

### Senescent macrophages accelerate VSMCs senescence

To explore the relationship between senescent macrophages and vascular aging in T2DM, IF staining was performed for α-SMA (vascular smooth muscle cell marker), CD31 (endothelial cell marker), F4/80 (macrophage marker), and p16 (senescence marker) in aortic samples from diabetic and healthy mice (Figure 2A). The findings indicated a significant increase in macrophage infiltration and elevated p16 expression levels in the aortas of diabetic mice (Figure 2B), even though some degree of senescence was observed in vascular smooth muscle and endothelial cells. To further explore the senescence phenotype of vascular macrophages in T2DM, TEM analysis of vessels was conducted. These results showed rounded mitochondria, a shallow matrix, and shortened or absent cristae in macrophages from the aortas of diabetic mice, indicative of senescence (Figures 2C and 2D).

*In vitro* studies on HG-induced senescence confirmed that macrophages exhibit senescent characteristics. Notably, HG levels significantly increased SA-β-Gal activity and upregulated senescence markers p16, p53, and p21, alongside a notable SASP (Figures 2E–2N). Additional TEM analysis demonstrated mitochondrial damage and autophagy impairment, evidenced by ruptured mitochondrial membranes, and a decreased count of autophagic vesicles (Figures 2O–2R). To further investigate the effects of HG on macrophages, Bafilomycin A1 (BafA1) was administered, a vesicular H^+^-ATPase inhibitor that impedes lysosomal function and prevents the entry of p62 and LC3B into the lysosome.^35^^,^^36^ We assessed changes in p62 and LC3 levels using WB to evaluate autophagic activity. The results indicated that HG exposure significantly impaired autophagic flux in macrophages, as evidenced by decreased overall LC3 expression. Additionally, following the addition of BafA1, a significant reduction in the autophagic flux of both p62 and LC3 was observed compared to the control group (Figure S2A). These results suggest accelerated macrophage senescence in diabetes, accompanied by a marked enhancement in SASP. Importantly, BMDMs-VSMCs co-culture system *in vitro* was established to further verified the impact of senescent macrophages on vascular aging in diabetes. The HG-induced senescent BMDMs significantly promoted SA-β-Gal activity in VSMCs and upregulated the expression of p53, p21, and p16, while downregulating the expression of PCNA (Figures 2S–2X).

### STING activation enhances macrophage senescence

**Figure 3:**
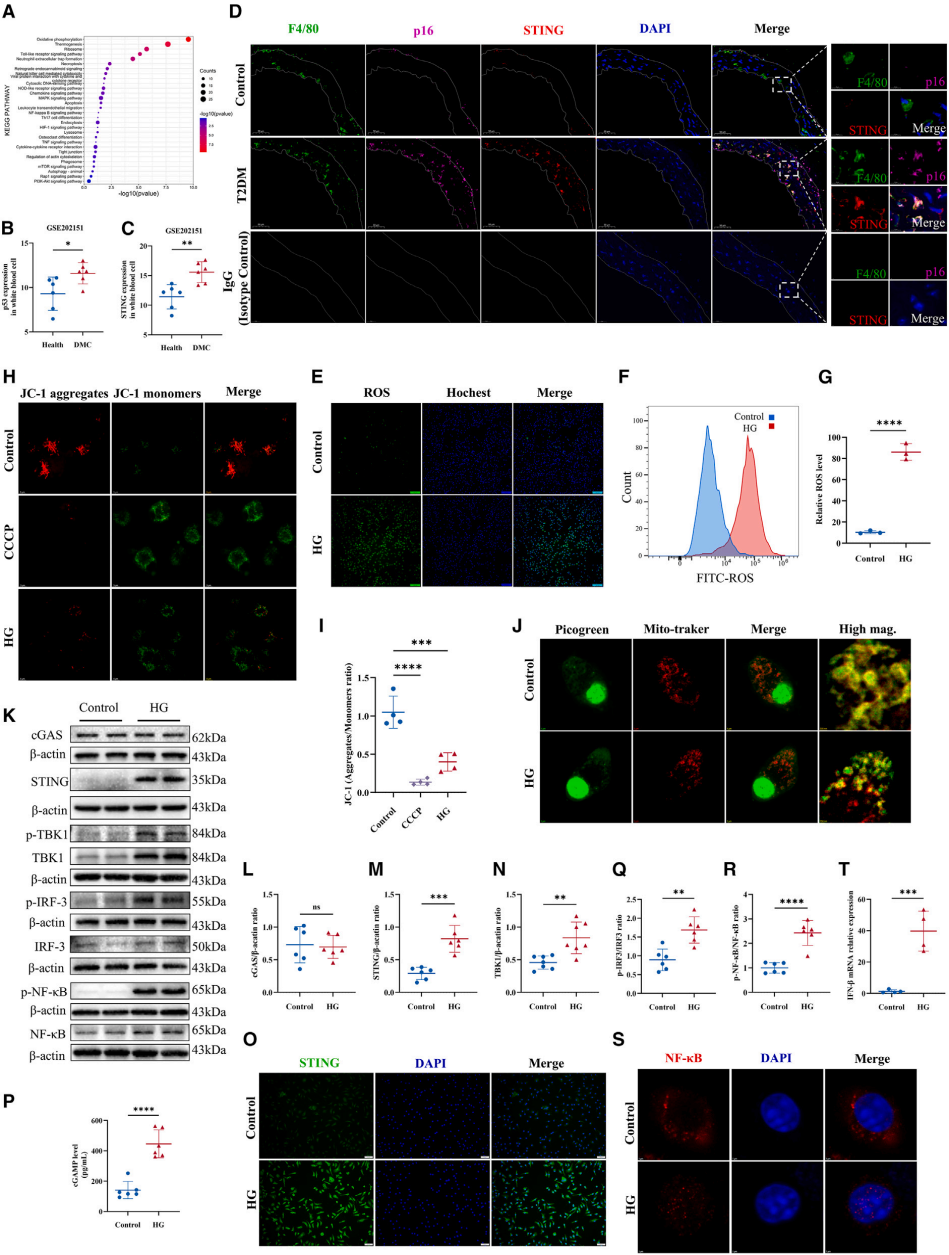
STING mediates macrophage senescence in diabetes mellitus (A) KEGG pathway analysis was performed using RNA-Seq data from the Database: GSE202151 dataset (Health, *n* = 6; DMC, *n* = 6). (B and C) mRNA levels of p53 and STING were measured in white blood cells from healthy controls (*n* = 6) and patients with type 2 diabetes with macrovascular complications (DMC; *n* = 6) using the same dataset. (D)Elevated STING levels were observed in p16^+^ macrophages of T2DM mice. Co-localized IF staining of p16, STING, and F4-80 was performed in blood vessels of diabetic mice, showing cells positive for all three markers. Scale bar: 50μm. (E–G) ROS levels were assessed through ROS staining and flow cytometry (*n* = 3). Scale bar: 100μm. (H and I) MMP changes were evaluated using JC-1 staining, with CCCP-treated BMDMs as a positive control (*n* = 4). Scale bar: 1μm. (J) BMDMs were labeled with the DNA-specific dye Picogreen and the mitochondrial probe Mitotracker Red. Scale bar: 1μm, 750nm, or 500nm. (K–N, Q, and R) Western blot analysis quantified the protein levels of cGAS, STING, *p*-TBK1, *p*-IRF-3, IRF-3, p-NF-κB, and NF-κB in BMDMs (*n* = 6). (O) The fluorescence intensity of STING was measured post-HG treatment. Scale bar: 50μm. (P) cGAMP levels in cell lysates were assessed (*n* = 6). (S) IF analysis confirmed the nuclear translocation of NF-κB. Scale bar: 1μm. (T) qRT-PCR was performed to measure the mRNA levels of IFN-β (*n* = 4). Data are presented as mean ± SD. ∗*p* < 0.05; ∗∗*p* < 0.01; ∗∗∗*p* < 0.001.

To explore the underlying mechanism of accelerated macrophage senescence in T2DM, RNA sequencing data (Database: GSE202151) from public databases were analyzed, focusing on white blood cells from healthy individuals and those with T2DM with macrovascular complications (DMC). Differential gene enrichment analyses highlighted an enhanced "cytosolic DNA-sensing pathway" (Figure 3A). Further, the upregulation of senescence markers p53 and STING genes in leukocytes from patients with DMC was observed compared to controls (Figures 3B and 3C). IF co-localization of STING, F4/80, and p16 showed more triple-positive cells in T2DM mice, suggesting that STING overexpression may contribute to macrophage senescence (Figure 3D).

To validate the upstream mechanism of cGAS-STING pathway activation, we investigated the potential for mitochondrial DNA leakage by performing ROS staining, conducting ROS flow cytometry, and assessing mitochondrial membrane potential (MMP) using JC-1 staining. Our results indicated that ROS levels were significantly elevated in macrophages cultured under HG conditions, as evidenced by increased green fluorescence intensity in both ROS staining and flow cytometry analysis (Figures 3E–3G). JC-1 staining assessed MMP changes, where high MMP results in JC-1 aggregation within the mitochondrial matrix, producing red fluorescence, and low MMP causes JC-1 to remain in its monomeric form, emitting green fluorescence.^37^^,^^38^ In our experiment, BMDMs were labeled with JC-1 dye, with CCCP serving as a positive control. The results demonstrated a significant reduction in MMP in HG-treated BMDMs (Figures 3H and 3I), indicating potential mitochondrial damage. To further confirm mtDNA leakage, we labeled BMDMs with the DNA-specific fluorescent dye Picogreen and the mitochondrial probe Mitotracker Red.^39^^,^^40^ This revealed mtDNA leakage from mitochondria in BMDMs under high-glucose conditions, providing a basis for cGAS-STING pathway activation (Figure 3J).

WB analysis showed that cGAS expression in BMDMs under HG conditions did not change significantly. However, levels of STING and TBK1 proteins were significantly increased (Figures 3K–3N). Following high-glucose treatment, STING fluorescence intensity also markedly increased (Figure 3O). Analysis of cell lysates revealed significantly elevated cGAMP levels in BMDMs treated with HG (Figure 3P), suggesting that HG may promote cGAS-STING pathway activity. Additionally, WB results indicated the increased phosphorylation of the downstream proteins IRF3 and NF-κB (Figures 3Q and 3R). IF analysis demonstrated that HG treatment promoted the nuclear translocation of NF-κB, indicating its functional activation (Figure 3S). Further qRT-PCR analysis revealed that mRNA levels of IFN-β were elevated in BMDMs cultured under HG conditions (Figure 3T). The primer sequences are shown in Table 1. Moreover, IF staining of F4/80, IRF3, and NF-κB in the aortas of diabetic mice showed that the number of IRF3 and NF-κB positive vascular macrophages in diabetic mice was significantly higher than in the control group (Figures S3A–S3D), suggesting that the activation of the cGAS-STING pathway in macrophages is closely related to macrophage senescence in the diabetic state.

**Table 1:** Primer oligonucleotide sequences used in this study

Gnen		Sequence (5' - 3')
*Sting*	Forward	GGTCACCGCTCCAAATATGTAG
	Reverse	CAGTAGTCCAAGTTCGTGCGA
*Tbk1*	Forward	GACATGCCTCTCTCCTGTAGTC
	Reverse	GGTGAAGCACATCACTGGTCTC
*Ifnb1*	Forward	GCCTTTGCCATCCAAGAGATGC
	Reverse	ACACTGTCTGCTGGTGGAGTTC
*Gapdh*	Forward	AAGGTCATCCCAGAGCTGAA
	Reverse	CTGCTTCACCACCTTCTTGA

Lysosomes serve as key negative regulators of cGAS-STING signal transduction, playing a critical role in the regulation of cGAS-STING signaling and its implications for human diseases.^41^ To further investigate the mechanisms underlying the increases in STING and TBK1 protein levels, BafA1 was used to inhibit lysosomal acidification and prevent the degradation of STING and TBK1 in lysosomes. The results indicated that the lysosomal degradation of STING was reduced under HG conditions (Figures S3E–S3G). IF staining of the lysosomal marker LAMP1 and STING showed decreased co-localization of the two signals under HG conditions (Figure S3H), suggesting the inhibition of STING degradation in lysosomes. In contrast, no significant changes were observed in TBK1 lysosomal degradation (Figures S3I and S3J). Additionally, the transcription levels of STING and TBK1 were assessed, showing significant increases for both, with TBK1’s transcription level increasing 4-fold compared to the control group (Figures S3K and S3L). In summary, the increased STING expression resulted partly from reduced lysosomal degradation and partly from elevated transcription levels, while the increase in TBK1 expression primarily arose from significant transcriptional upregulation.

**Figure 4:**
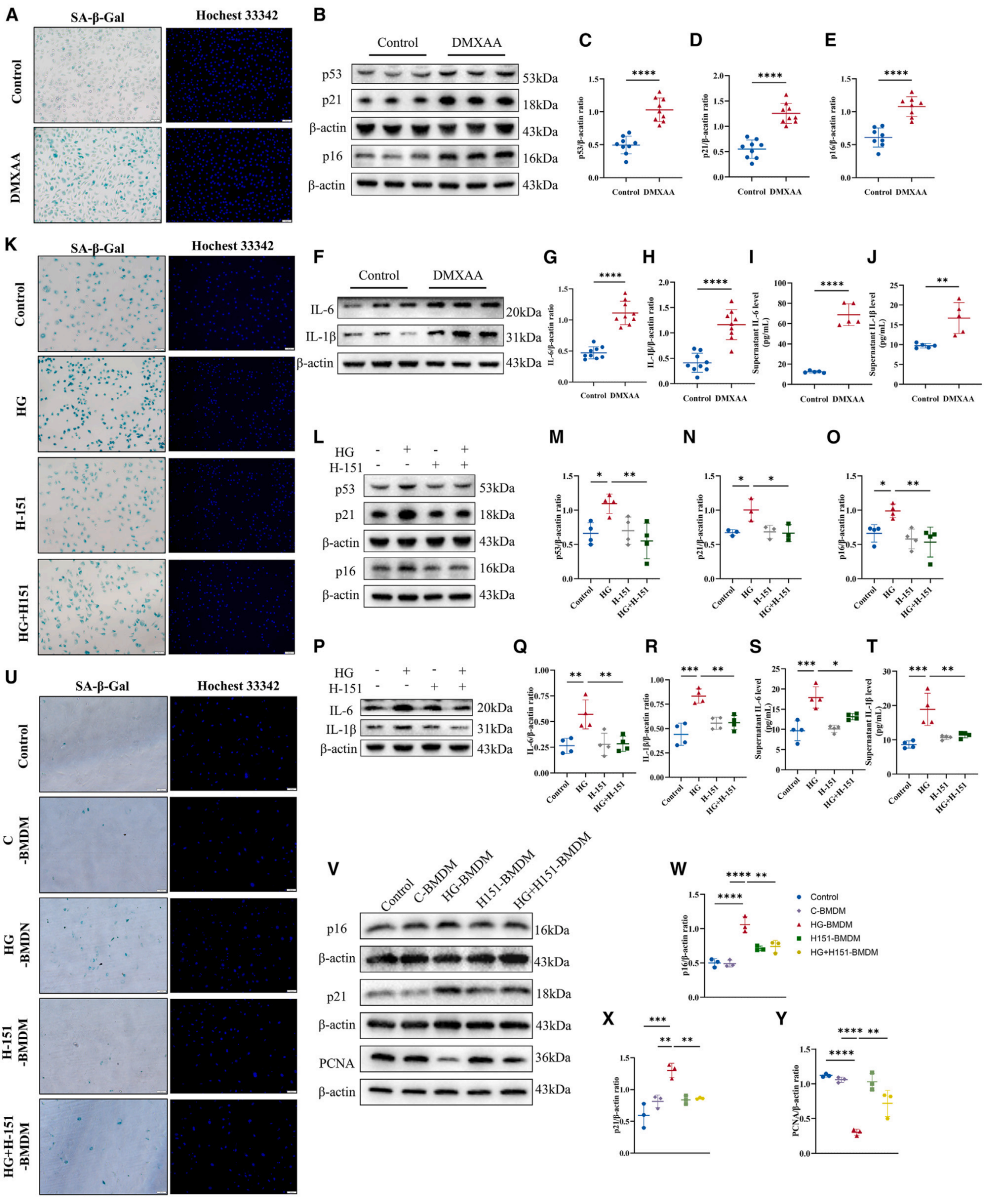
STING promotes senescence of macrophages exposed to HG (A) SA-β-Gal staining highlighted senescent cells, displaying green in activated senescent BMDMs, with nuclei stained using Hochest33342. (scale bar: 50μm). (B–H) p53, p21, p16, IL-6, and IL-1β protein levels were quantified by immunoblotting, using β-actin as a loading control. (n = 8–9). (I and J) BMDMs were treated with DMXAA for 72 h, and the concentrations of IL-6 and IL-1β in the supernatants were measured using ELISA (*n* = 5). (K) SA-β-Gal staining was used again to highlight senescent cells, showing green in activated senescent BMDMs, with nuclei stained using Hochest33342. (scale bar: 50μm). (L–R) p53, p21, p16, IL-6, and IL-1β protein levels were quantified by immunoblotting, using β-actin as a loading control. (*n* = 4). (S and T) The concentrations of IL-6 and IL-1β in the supernatants were measured using ELISA (*n* = 4). (U) SA-β-Gal staining highlighted senescent cells with nuclei counterstained using Hoechst 33342 (scale bar: 100 μm). (V–Y) p21, p16, and PCNA protein levels were quantified by immunoblotting, using β-actin as a loading control (*n* = 3). Data are presented as mean ± SD. ∗*p* < 0.05; ∗∗*p* < 0.01; ∗∗∗*p* < 0.001.

The influence of HG on macrophage STING activation was further investigated by utilizing the STING activator DMXAA and the inhibitor H-151. Consistent with HG effects, DMXAA treatment led to increased SA-β-Gal activity (Figure 4A), as well as the upregulation of p53, p21, and p16 in BMDMs (Figures 4B–4E). Subsequent analysis revealed increased protein levels of IL-6 and IL-1β in DMXAA-treated BMDMs, along with elevated concentrations of these cytokines in the cell supernatants (Figures 4F–4J). Conversely, co-treatment with H-151 reversed the upregulation of SA-β-Gal, p53, p21, p16, IL-6, and IL-1β in HG-exposed BMDMs (Figures 4K–4T), indicating that H-151 can mitigate HG-induced macrophage senescence. To further validate the impact of H-151 on HG-induced macrophage senescence and its effects on vascular smooth muscle cells, we co-cultured H-151-treated, HG-exposed macrophages with vascular smooth muscle cells. Assessment through Senescence-associated SA-β-Gal staining and WB analysis of p16, p21, and PCNA confirmed that H-151 postponed macrophage senescence induced by HG, thereby reducing the senescence of vascular smooth muscle cells (Figures 4U–4Y).

To verify the effect of the STING inhibitor on diabetes-related macrophage senescence, we established a diabetic mouse model through the intraperitoneal injection of streptozotocin (STZ) and treated the mice with H-151. Following STZ injection, the blood glucose levels in the mice significantly increased, meeting the diabetic standard (Figure S4A). The results of the glucose tolerance test after intraperitoneal injection demonstrated impaired glucose tolerance in both the STZ group and the STZ+H-151 group (Figure S4B). We conducted Western blot (WB) analysis on the peripheral blood mononuclear cells (PBMCs) from the mice to detect the protein levels of p53, p21, and p16 in the PBMCs (Figures S4C–S4F), and the results indicated that H-151 effectively alleviated macrophage senescence compared to STZ mice. Considering the abundant distribution of macrophages in the spleen, we performed IF staining for p16 and F4/80 on the spleens of the four groups of mice. The results showed that the p16 levels in macrophages from the STZ group were significantly increased, while H-151 treatment effectively reduced this condition (Figures S4G and S4H). Additionally, we conducted similar IF staining on the aortas of the four groups of mice, yielding similar findings (Figures S4I and S4J).

### Autophagy flux is inhibited by prolonged STING activation

**Figure 5:**
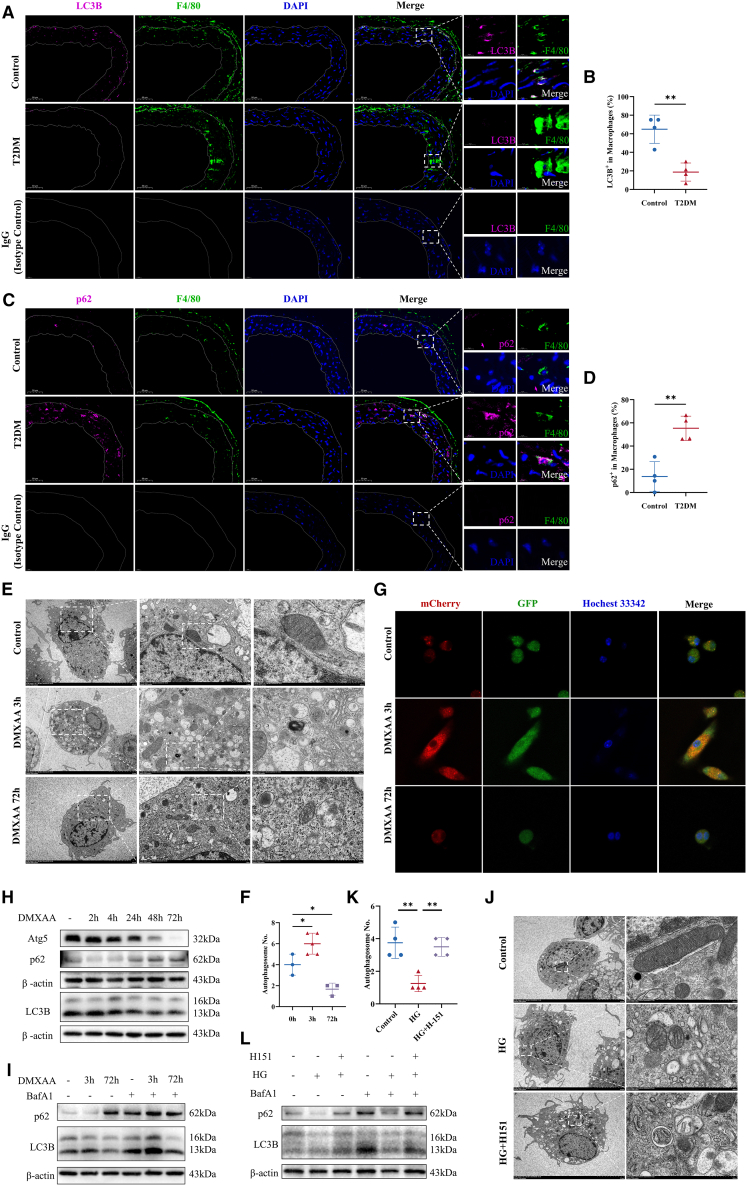
Autophagy pathway inhibited by aberrant STING activation (A–D) Representative IF images of LC3B and p62 in the aortic endothelium of the indicated mouse groups (*n* = 4). (scale bar: 50μm). (E and F) Representative TEM images of autophagosome. Data are presented as a dot plot of the number of autophagosomes (n = 3–5; scale bar: 5μm or 500nm). (G) To investigate autophagy in BMDMs, mCherry-GFP-LC3B adenoviruses were transfected. Upon autophagy activation, mCherry-GFP-LC3B aggregates on the autophagosome membrane and appears as a yellow spot. (H) Changes in autophagy in BMDMs following intervention with the STING agonist DMXAA were assessed p62, LC3B, and Atg5 protein levels were measured by WB. (I) BMDMs were treated with DMXAA and subsequently with BafA1 3 h prior to sample collection to enhance autophagy detection; LC3B and p62 protein levels were evaluated by WB. (J and K) TEM was used to observe autophagosomes in macrophages treated with H-151 under HG conditions (*n* = 4). (L) Western blot analysis was conducted to assess the levels of P62 and LC3B in macrophages after H-151 treatment. Data are presented as mean ± SD. ∗*p* < 0.05; ∗∗*p* < 0.01; ∗∗∗*p* < 0.001.

Autophagy inhibition, a characteristic of aging,^42^^,^^43^ and the complex role of STING in this process,^24^^,^^44^^,^^45^ will be explored in future studies. In diabetic mice, a reduction in LC3B expression and an increase in p62 expression were noted in macrophages from aortas (Figures 5A–5D). These changes indicate that macrophage senescence in diabetes may be partially due to autophagy inhibition.

To further explore the interaction between STING and autophagy, autophagy flux was measured in macrophages exposed continuously to DMXAA. TEM analysis confirmed a decrease in autophagic vesicles after 72 h, despite an initial increase post 3-h intervention (Figures 5E and 5F). Adenoviral AdPlus-mCherry-GFP-LC3B detection showed a significant reduction in autophagic vesicles after 72 h, falling below control levels (Figure 5G). Although autophagy was initially activated, p62 accumulation and a decrease in LC3B and Atg5 levels were observed after 72 h (Figure 5H). Using BafA1 to observe changes in autophagy, autophagy flux was activated after 3 h of DMXAA treatment, evidenced by increased lysosomal degradation of p62 and LC3 (Figure 5I). However, autophagy flux was significantly reduced after 72 h of treatment, marked by p62 accumulation and decreased LC3B levels (Figure 5I). To further clarify the role of STING in autophagy, we intervened with H-151 in macrophages exposed to HG and observed its effects on autophagy in aging macrophages. TEM results showed that H-151 reversed the reduction of autophagic vesicles caused by HG (Figures 5J and 5K). Additionally, WB analysis showed that H-151 enhanced the degradation of autophagy substrates P62 and LC3B in lysosomes of macrophages exposed to HG (Figure 5L). Furthermore, our study found that DMXAA exacerbated the reduction in the number of autophagic vesicles and the lysosomal degradation of P62 and LC3B caused by HG (Figures S5A–S5C).

### STING inhibits autophagy flux by phosphorylating ULK1 in senescent macrophages

**Figure 6:**
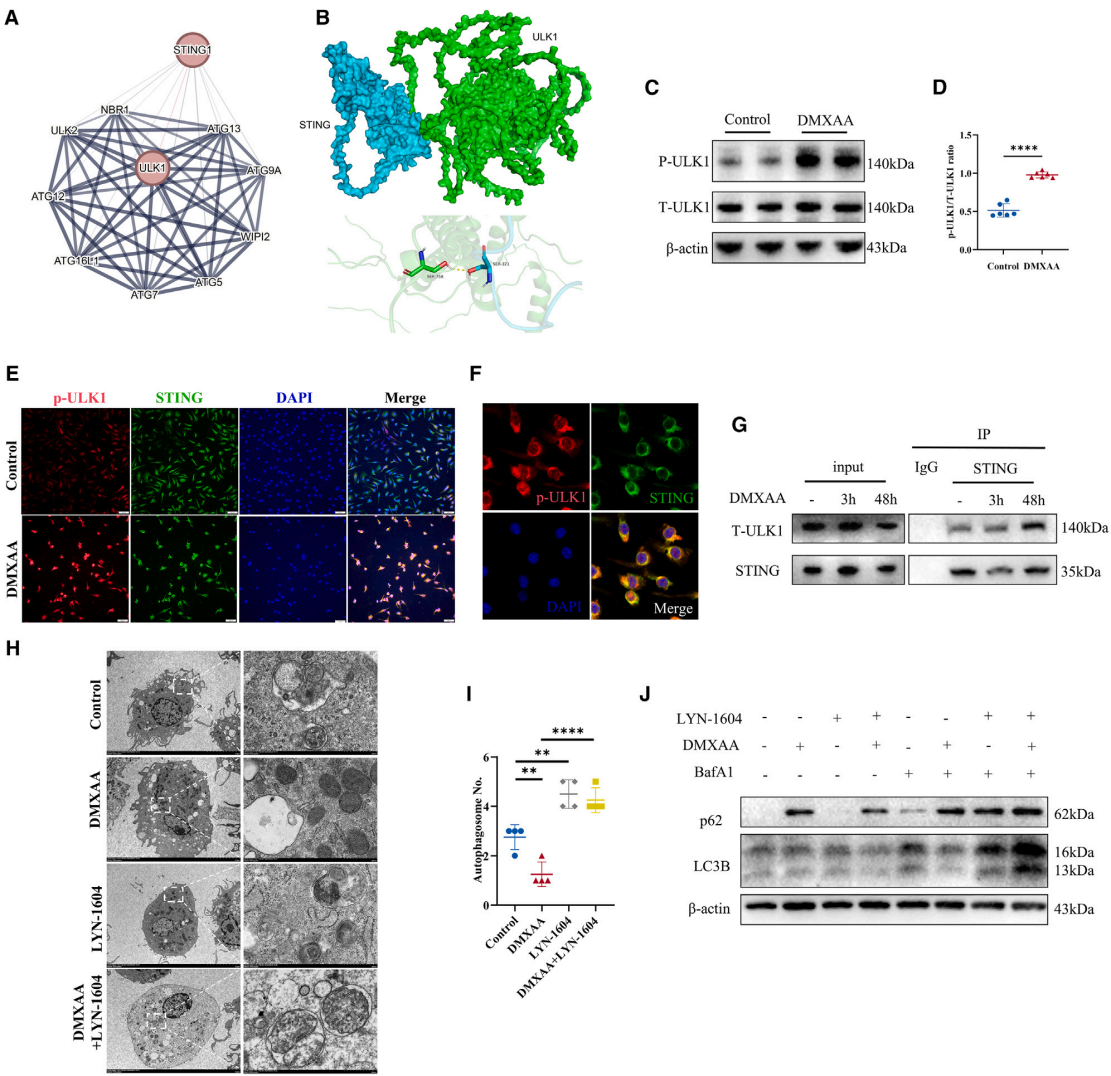
STING promotes phosphorylation at the S757 site of ULK1 (A) The protein-protein interaction network illustrates the interaction between STING and ULK1. (B) Protein-protein docking analysis was conducted to predict the potential interaction between STING and ULK1. (C and D) ULK1 and *p*-ULK1 were detected by Western blot. Relative levels of *p*-ULK1 protein to ULK1 protein were quantified. (*n* = 6). (E) Representative IF staining illustrated the co-localization of STING and *p*-ULK1 in BMDMs. (F) IF staining was used to co-localize STING and ULK1 protein expression in BMDMs. Note the bright STING staining in cells co-localized with prominent ULK1 staining. (G) Coimmunoprecipitation of STING and ULK1 in BMDMs was conducted. Lysates were immunoprecipitated with an anti-STING antibody. Precipitates and lysates were analyzed by WB using anti-ULK1 and anti-STING antibodies. (H and I) TEM was employed to observe the number of autophagosomes in macrophages treated with LYN-1604 under conditions of STING activation. (J) Western blot analysis was performed to assess the levels of P62 and LC3B in macrophages treated with LYN-1604 under STING activation conditions. Data are presented as mean ± SD. ∗*p* < 0.05; ∗∗*p* < 0.01; ∗∗∗*p* < 0.001.

Protein-protein interaction analysis suggested a strong association between STING and ULK1 signaling (Figure 6A). Additionally, we employed protein-protein docking to predict a potential interaction between STING and ULK1 (Figure 6B). Understanding ULK1 regulation, an autophagy-initiating kinase, is vital for elucidating autophagy regulatory mechanisms.^46^ Sensors of cellular energy metabolism inhibit autophagy activation by phosphorylating ULK1 at Ser 757, while reduced phosphorylation at this site initiates autophagy.^47^^,^^48^ Investigations into STING-mediated autophagy flux inhibition in macrophages showed it correlates with the S757 phosphorylation of ULK1. Western blot analysis revealed increased S757 phosphorylation after 72 h of DMXAA treatment (Figures 6C and 6D). IF double-labeling of *p*-ULK1 (S757) with STING indicated enhanced ULK1 phosphorylation with prolonged DMXAA activation (Figure 6E). To confirm potential protein-protein interactions between STING and ULK1, laser confocal microscopy and Co-IP analysis were utilized. The results confirmed interactions between STING and ULK1, and laser confocal microscopy demonstrated co-localization of STING with *p*-ULK1 (Figures 6F and 6G). To further confirm the critical role of ULK1 phosphorylation in the STING-mediated inhibition of autophagic flux, we introduced the ULK1 agonist LYN-1604. LYN-1604 binds to the activation sites of ULK1 (LYS50, LEU53, and TYR89), upregulating *p*-ULK1 (ser317) and downregulating *p*-ULK1 (ser757), thereby enhancing autophagy.^49^ Transmission electron microscopy results demonstrated that LYN-1604 alleviated the reduction in autophagic flux induced by STING activation, as evidenced by the increased number of autophagosomes (Figures 6H and 6I). Additionally, with the addition of BafA1, we observed that LYN-1604 could reverse the DMXAA-induced decrease in autophagic substrate P62 and the reduction in LC3B conversion (Figure 6J). These findings underscore the importance of ULK1 in STING-induced autophagy regulation and illuminate the mechanisms by which ULK1 phosphorylation modulates autophagic flux.

## Discussion

Herein, we propose a mechanism by which STING-mediated macrophage senescence contributes significantly to diabetic vascular aging. Additionally, our research indicates that STING activation leads to phosphorylation at the ULK1 S757 site, suppressing autophagy and accelerating macrophage senescence. These insights suggest that targeting STING inhibition could delay macrophage senescence, thereby mitigating diabetic vascular aging and reducing the risk of related cardiovascular diseases.

Undoubtedly, diabetes represents a state of premature aging.^4^^,^^50^ However, targeting specific diabetic complications does not address the root cause of the disease, thus, identifying the common pathogenesis of these comorbidities remains a critical focus of current research.^51^^,^^52^ In this context, early detection and effective management of vascular aging are crucial to addressing the growing problem of diabetic complications.^6^ In this study, we comprehensively and objectively evaluated vascular aging in T2DM, assessed vascular structure, function, and protein markers, and confirmed significant vascular senescence.

Given the inherently contagious nature of senescence, our study aimed to elucidate the origins of vascular senescence in diabetes.^53^^,^^54^ Initially, senescent cells in tissues and organs trigger a chronic, low-grade inflammatory response in adjacent cells through the secretion of SASPs, leading to widespread tissue or organ senescence and dysfunction. Recent research using IF staining confirmed prominent p16 expression in macrophages within diabetic periodontal lesions.^14^ Similarly, high levels of senescence were noted in both endothelial cells and macrophages of diabetic mice kidneys, characterized by a SASP.^55^ Our analysis of vascular tissues from diabetic mice primarily identified senescence in macrophages. Considering mitochondrial damage as a hallmark of senescence, we used TEM to verify macrophage senescence, revealing damaged mitochondrial membranes and loss of mitochondrial cristae. To further support that macrophage senescence contributes to diabetic vascular senescence, we implemented an HG-induced macrophage senescence model in co-culture with vascular smooth muscle cells. Despite these efforts, our study remains limited to *in vitro* assessments of the interaction between macrophage senescence and vascular senescence. Notably, we have not yet applied *in vivo* methods, such as senescent macrophage adoptive transfer or bone marrow transplantation, to definitively determine whether macrophage senescence is the primary cause of vascular senescence in diabetes.

This study employed a cross-sectional design, which lacks the observation of dynamic changes, thus presenting limitations compared to longitudinal studies. To enhance the depth of longitudinal research, we plan to utilize spatiotemporal multi-omics technologies in future investigations to comprehensively explore the spatiotemporal evolution of diabetic vascular aging. By integrating spatial transcriptomics with immunofluorescence staining, we will be able to observe the gene expression patterns of macrophages and vascular smooth muscle cells within the spatial context of the tissue, revealing their local interactions in the diabetic microenvironment. Additionally, flow cytometry will be used to isolate F4/80+ CD11b+ double-positive cells for single-cell RNA sequencing, enabling us to further analyze transcriptional characteristics at the single-cell level and accurately capture changes in the functional status of macrophages during senescence. The combination of these two technologies will provide a multidimensional and spatiotemporally integrated perspective, thereby deepening our understanding of how macrophage senescence drives diabetes-related vascular aging.

Cellular senescence is triggered by various factors, including DNA damage, telomere shortening, oxidative stress, and oncogenes, all of which directly or indirectly impact DNA integrity.^56^ Notably, STING is activated by cGAMP produced by cGAS, a DNA receptor. STING plays a significant role in obesity-induced diabetes,^20^ myocardial infarction,^19^ and chronic inflammatory diseases.^57^^,^^58^ Thus, STING activation may contribute to premature aging in patients with diabetes. We confirmed STING activation in p16-positive macrophages within the vasculature of T2DM mice and observed its expression in HG-induced senescent macrophages *in vitro*. We also validated that sustained STING activation induces macrophage senescence using the agonist DMXAA, consistent with recent research.^59^ Despite diabetic vascular complications being a leading cause of mortality among diabetics, mechanism-based therapeutics are scarce, as stringent glycemic control fails to eradicate vascular damage.^60^^,^^61^ We identified STING as a potential therapeutic target and used H-151, a potent and selective inhibitor, to attenuate STING activation in HG-treated macrophages. Our findings revealed that H-151^62^^,^^63^ mitigated HG-induced expression of senescence markers, including β-galactosidase, p16, p53, p21, and SASP. Consequently, targeting STING inhibition represents a promising therapeutic approach for managing macrophage senescence.

To elucidate the regulatory mechanisms governing the upregulation of STING and TBK1 in high glucose conditions, we investigated lysosomal degradation^41^ and transcriptional activation as potential contributory factors. Utilizing BafA1 to inhibit lysosomal acidification, we observed a significant reduction in STING degradation under high glucose conditions, whereas TBK1 degradation remained unchanged. Transcriptional analysis indicated that both proteins were markedly upregulated, with TBK1’s transcriptional levels increasing approximately 4-fold relative to the control group. The enhanced expression of STING can be attributed to both inhibited lysosomal degradation and augmented transcriptional activity, while the upregulation of TBK1 predominantly arises from transcriptional activation. These findings significantly advance our understanding of the cGAS-STING pathway’s role in the pathophysiology of diabetes.

STING proteins' ability to induce autophagy is a fundamental function that preceded the regulation of IFN-1 transcriptional expression in evolutionary history.^24^ Upon cGAMP binding and activation, STING translocates from the endoplasmic reticulum to the ERGIC, facilitating LC3 recruitment and lipidation via a WIPI2-dependent mechanism, leading to autophagic vesicle formation.^24^^,^^44^^,^^64^^,^^65^ULK1-initiated canonical autophagy and STING-mediated autophagy facilitate the accumulation of double-membrane vesicles and single-membrane vesicles, respectively, exhibiting a parallel nature of these two pathways.^66^^,^^67^ Furthermore, from the perspective of competitive binding with WIPI2, these two autophagic pathways exhibit mutual inhibition. In classical autophagy, the mechanism bridging the initial activation phase, facilitated by the ULK1 and PI3KC3 complexes that promote PI3P synthesis, and the extension phase, which involves the lipidation of the Atg8 protein family, has remained a pivotal yet unresolved issue in cellular autophagy research. However, the identification of WIPI2 has provided a definitive answer to this enduring scientific inquiry. WIPI2 acts as a crucial bridging molecule, binding to PI3P and recruiting the ATG16L1-ATG5-ATG12 complex through its interaction with ATG16L1, thereby efficiently mediating the lipidation of the ATG8 protein family at PI3P-enriched autophagic precursors.^65^^,^^68^ Interestingly, despite their critical role in initiating classical autophagy, protein complexes such as ULK1 and PI3KC3 are not involved in STING-mediated autophagy, whereas WIPI2 serves as a common factor in both pathways. Upon activation, STING moves from the endoplasmic reticulum to the ER-Golgi intermediate compartment (ERGIC), forming an autophagosome precursor. STING then directly recruits WIPI2 to this precursor via protein-protein interactions, facilitating the recruitment of the ATG16L1-ATG5-ATG12 complex. This promotes the lipidation of the ATG8 protein family and the elongation of the autophagosome precursor.^25^ Impaired autophagy is a significant indicator of cellular senescence.^69^ Our research using the STING agonist DMXAA to intervene persistently in macrophages revealed that senescence in macrophages was accompanied by decreased autophagy levels. Consequently, we hypothesized that prolonged STING activation might promote the phosphorylation of ULK1 at the S757 site, suppressing classical autophagy and potentially explaining the cellular senescence observed in macrophages. This hypothesis was supported by a study on mice undergoing aortic narrowing surgery.^26^ STING may inhibit autophagy by enhancing the phosphorylation of ULK1’s S757 site, thus preventing pressure overload-induced cardiac remodeling.^26^ Our study also revealed a potential protein-protein interaction between ULK1 and STING through the application of IF double labeling and Co-IP.

Notably, several studies have indicated that STING initiates autophagy,^24^^,^^44^^,^^70^ which contrasts with our observations. However, several explanations exist for this discrepancy. Firstly, in terms of experimental methods, these studies primarily examined autophagy promotion during the first 12 h of STING activation, overlooking its effects over extended periods. In contrast, our experiments using autophagy flux detection, electron microscopy, and Western blot demonstrated that while STING activation induces autophagy in the short term (3 h), its prolonged activation (72 h) results in autophagy inhibition. Secondly, considering the relationship between STING and aging, autophagy inhibition is a characteristic of senescence, and STING is active in older individuals.^23^^,^^71^^,^^72^ Therefore, we propose a potential causal relationship between autophagy suppression and prolonged STING activation. Finally, although the interaction between STING and autophagy is complex, research has identified competitive inhibition between ULK1-mediated classical autophagy and STING-mediated non-classical autophagy for WIPI2, highlighting the need for a more comprehensive understanding of how STING regulates autophagy.

### Limitations of the study

There are noteworthy limitations to this study. First, given the prevalence of STING in diabetic complications, potentially as an early indicator, further validation in individuals with diabetes is crucial for transitioning from basic to clinical research. Second, although our findings suggest that macrophage senescence accelerates vascular aging, additional studies across various stages of diabetic disease are necessary to clarify the temporal aspects of diabetic vascular aging. Lastly, while our research indicates that inhibiting STING reduces macrophage senescence in T2DM, its impact on natural aging remains unclear, requiring more extensive studies to assess the applicability of our findings.

## Resource availability

### Lead contact

Further information and requests for resources and reagents should be directed to and will be fulfilled by the lead contact, Shi Tai (taishi2017@csu.edu.cn).

### Materials availability

This study did not generate new unique reagents. Any additional analysis information for this work is available by request to the lead contact.

### Data and code availability

• All data reported in this article will be shared by the lead contact upon request.
• This article does not report the original code.
• Any additional information required to reanalyze the data reported in this article is available from the lead contact upon request.

## Acknowledgments

The authors are very grateful to Yuying Zhou and Jiabao Zhou for their technical assistance. This work was supported by the 10.13039/501100001809National Natural Science Foundation of China (82271601), the Fundamental Research Funds for the Central Universities of Central South University (Grant No. 930), and the 10.13039/501100004735Natural Science Foundation of Hunan Province (2023JJ30780 and 2022JJ40683).

## Funding

This research was supported by the National Natural Science Foundation of China (82271601), the Fundamental Research Funds for the Central Universities of Central South University (Grant No.930), and the Natural Science Foundation of Hunan Province (2023JJ30780 and 2022JJ40683).

## Author contributions

Huiqing Ding: Conceived and designed the experiments; analyzed and interpreted the data; wrote the article; performed the experiments.

Quan Zhang, Rukai Yang: Performed the experiments, analyzed and interpreted the data.

Liyao Fu, Hejun Jiang, Qingyi Zhu: Contributed reagents, materials, analysis tools or data.

Shi Tai: Conceived and designed the experiments; revised the article; analyzed and interpreted the data; contributed reagents, materials, analysis tools or data.

## Declaration of interests

The authors declare that they have no competing interests.

## STAR★Methods

### Key resources table

**Table:** 

REAGENT or RESOURCE	SOURCE	IDENTIFIER
**Antibodies**		

CDKN2A/p16 antibody（F-12）	Santa Cruz Biotechnology	Cat#sc-1661; RRID：AB_628067
p21 Polyclonal antibody	Proteintech	Cat#28248-1-AP; RRID：AB_2881097
p53 Monoclonal antibody	Proteintech	Cat#60283-2-IG; RRID：AB_2881401
STING (D2P2F) Rabbit mAb	Cell Signaling Technology	Cat#13647S; RRID：AB_2799947
TBK1/NAK (E8I3G) Rabbit mAb	Cell Signaling Technology	Cat#38066S;RRID：AB_2827657
Phospho-TBK1/NAK (Ser172) (D52C2) XP® Rabbit mAb	Cell Signaling Technology	Cat#5483S; RRID：AB_10693472
LC3B-Specific Polyclonal antibody	Proteintech	Cat#18725-1-AP; RRID：AB_2137745
SQSTM1/p62 (D1Q5S) Rabbit mAb	Cell Signaling Technology	Cat#39749; RRID：AB_2943237
Phospho-ULK1 (Ser757) (D7O6U) Rabbit mAb	Cell Signaling Technology	Cat#14202S; RRID：AB_2665508
ULK1 Monoclonal antibody	Proteintech	Cat#68445-1-IG; RRID：AB_3085157
IL-6 Polyclonal antibody	Proteintech	Cat#21865-1-AP; RRID：AB_11142677
IL-1 beta Polyclonal antibody	Proteintech	Cat#26048-1-AP; RRID：AB_2880351
PCNA (D3H8P) XP® Rabbit mAb	Cell Signaling Technology	Cat#13110; RRID：AB_2636979
cGAS (D3O8O) Rabbit mAb	Cell Signaling Technology	Cat#31659; RRID：AB_2799008
IRF-3 (D83B9) Rabbit mAb	Cell Signaling Technology	Cat#4302; RRID：AB_1904036
Phospho-IRF-3 (Ser396) (4D4G) Rabbit mAb	Cell Signaling Technology	Cat#4947; RRID：AB_823547
NF-κB p65 (D14E12) XP® Rabbit mAb	Cell Signaling Technology	Cat#8242; RRID：AB_10859369
Phospho-NF-κB p65 (Ser468) Recombinant antibody	Proteintech	Cat#82335-1-RR; RRID：AB_3083091
F4/80 Polyclonal antibody	Proteintech	Cat#28463-1-AP; RRID：AB_2881149
Biotin-conjugated Goat Anti-Mouse IgG(H+L)	Proteintech	Cat#SA00004-1; RRID：AB_2890900
Biotin-conjugated Goat Anti-Rabbit IgG(H+L)	Proteintech	Cat#SA00004-2; RRID：AB_2890944

**Chemicals, peptides, and recombinant proteins**		

Streptozocin	Sigma	Cat#S0130
H-151	MCE	Cat#HY-112693
DMXAA	MCE	Cat#HY-10964
Tween-80	Sigma	Cat#P1754
Mouse M-CSF	PeproTech	Cat#AF-315-02-50UG
mononuclear cell isolation solution	DAKEWE	Cat#DKW33-R0100

**Critical commercial assays**		

Masson’s Trichrome Staining Kit	BIOSSCI Biotech	Cat#BP028
Verhoeff’s Van Gieson Staining Kit	BIOSSCI Biotech	N/A
SA-β-Gal Staining Kit	Beyotime	Cat#C0602
JC-1 Staining Kit	Beyotime	Cat#C2003S
ROS Assay Kit	Beyotime	Cat#S0035S
Mito-Tracker Red	Beyotime	Cat#C1034
Picogreen	Yeasen	Cat#12641ES01
cGAMP ELISA Kit	Cayman Chemical	Cat#501700
IL-6 ELISA Kits	Jianglai	Cat#JLW20268
IL-1β ELISA Kits	Jianglai	Cat#JLW18442
rProtein A/G	MCE	Cat#BK0004-02
TRIzol	Invitrogen	Cat#15596026
RevertAid First Strand cDNA synthesis kit	Thermo Fisher Scientific	Cat#K1622
SYBR Green Supermix	Bio-Rad	Cat#1725124

**Experimental models: Cell lines**		

BMDMs	In-house laboratory	N/A
VSMCs	In-house laboratory	N/A

**Experimental models: Organisms/strains**		

C57BL/6 Mice (Wild-Type)	In-house laboratory	N/A

**Recombinant DNA**		

mCherry-GFP-LC3B Adenoviral Vector	Beyotime	Cat#C3012

**Software and algorithms**		

Prism 9	GraphPad	https://www.graphpad.com
Image J	NIH	https://imagej.nih.gov/

**Other**		

Laser Confocal Microscope	Carl Zeiss	LSM710
High-fat feeds	Research diet	Cat#D12492
Accu-Chek Aviva glucose analyzer	Rotkreuz	Roche Diagnostics
non-invasive computerized tail-cuff system	Kent Scientific	CODA 6
transmission electron microscope	HITACHI	HT7700
The RNA-Seq data of white blood cells from healthy controls and patients with type 2 diabetes and macrovascular complications.	Gene Expression Omnibus	GSE202151

### Experimental model and study participant details

#### Animals

A high-fat diet (HFD)^73^^,^^74^ and low-dose streptozotocin (STZ, 45 mg/kg) were used to induce T2DM in C57BL/6 mice.^75^^,^^76^ Initially, 2-month-old C57BL/6 mice were used to induce type 2 diabetes mellitus (T2DM) with a high-fat diet (HFD, D12492, Research Diet) and low-dose streptozotocin (STZ, 45 mg/kg). Mice were randomly assigned to the diabetes group and the control group at the start. Initially, the mice were fed an HFD for two months, followed by three consecutive days of intraperitoneal STZ injections dissolved in sterile citrate buffer. One week after the STZ injection, mice with blood glucose levels ≥16.7 mmol/L were diagnosed with T2DM. The diabetes group continued to receive the HFD for an additional five months, while the control group was fed a normal diet (ND) and received citrate buffer injections. The number of mice per group was *n*=6 or 7. Body weight was monitored weekly, and blood glucose levels were measured monthly using tail-tip sampling and a glucometer. The validity of the diabetes model was confirmed with an intraperitoneal glucose tolerance test (IPGTT; 1 mg/kg body weight) performed one week after the STZ injection. Vascular aging was assessed through ultrasonography, measuring aortic thickness, pulse wave velocity, and non-invasive blood pressure prior to sample collection.

To assess the *in vivo* effects of the STING inhibitor on macrophage senescence, we established a diabetic mouse model through continuous intraperitoneal injections of STZ at a dose of 50 mg/kg in 0.1 mol/L citrate buffer (pH 4.5) for 5 days.^77^ Mice were considered as diabetic if their random blood glucose levels exceeded 16.7 mmol/L. As previously described,^78^ two weeks following the STZ injections, mice treated with H-151 (HY-112693, MCE) received an intraperitoneal injection of 750 nmol per mouse in 200 μl of PBS containing 10% Tween-80. At the start of the experiment, all mice were randomly assigned to one of four groups: Control, STZ, H-151, and STZ+H-151. The sample size for each group was n=4-5. All mice continued on their respective diets for four weeks before isolation of blood vessels, spleens, and PBMCs for further analysis.

Mice were housed under controlled conditions (22 ± 2°C) with a 12-hour light/dark cycle and had *ad libitum* access to food and water. All procedures were approved by the CSU Animal Care and Use Committee.

### Method details

#### Fasting blood glucose test and intraperitoneal glucose tolerance test

Weekly blood glucose measurements were taken after a 14-hour fast using an Accu-Chek Aviva glucose analyzer (Roche Diagnostics, Rotkreuz, Switzerland). After 2 months, an IPGTT was administered to a subset of mice (n = 6-7 per group). Following a 14-hour fast, glucose (1 mg/kg body weight) was injected, and tail vein blood samples were collected at 0, 15, 30, 60, and 120 minutes post-injection.

#### Non-invasive blood pressure measurements

Systolic, diastolic, and mean blood pressures were measured in awake mice using a non-invasive computerized tail-cuff system (CODA 6, Kent Scientific, USA). During a 3-day acclimatization period, the animals were trained with the tail-cuff device. Blood pressure measurements were conducted at least 20 times per mouse until a steady state was achieved, with final values calculated from an average of the 10 closest consecutive readings.

#### Pulse wave velocity (PWV) assessment

After anesthetizing the mice, they were positioned supine, and the paws were attached to the electrode on an ECG plate. A 20 MHz Doppler probe was used to measure the velocity of the aortic arch at a depth of 2-4 mm. Measurements were taken 25-30 mm distal from the abdomen, with the probe angled toward the heart at a depth of 2-3 mm. The aortic pulse wave was calculated by dividing the distance (25-30 mm) by the difference in arrival times of the velocity pulse timed with respect to the ECG.

#### Immunohistochemistry

Immunohistochemical analysis was performed to assess the expression of p53 and p21 in vascular tissues. Sections were blocked for 30 minutes at room temperature (20°C-25°C) and then incubated overnight at 4°C with primary antibodies against p21 (Proteintech, #10355-1-AP; 1:400) and p53 (Proteintech, #10442-1-AP; 1:100). Isotype control antibodies were also used for appropriate comparison under the same experimental conditions to assess non-specific background signal. Subsequently antibodies (Proteintech, #SA00004-1 and #SA00004-2; 1:100) were applied for 30 minutes at room temperature. DAB solution was used for development, followed by hematoxylin restaining, and sections were visualized under an Olympus microscope (Japan). Quantitative analysis was conducted using Image J (National Institutes of Health), averaging values across four quadrants.

#### Immunofluorescence

Immunofluorescence was utilized to detect macrophage senescence markers and autophagy markers in mouse aorta sections. Sections were incubated overnight at 4°C with antibodies targeting F4/80 (Proteintech, #28463-1-AP; 1:200), p16 (Santa Cruz Biotechnology, #sc-1661; 1:200), STING (CST, #13647S; 1:200), LC3B (Proteintech, #18725-1-AP; 1:200), and p62 (CST, #39749; 1:100). Isotype control antibodies were also used under the same experimental conditions to assess non-specific background staining. After rinsing, sections were incubated with a secondary antibody solution diluted in TBST for 45 minutes at 37°C. Tyramine salt solution was added dropwise, incubated for 10 minutes at room temperature shielded from light, and eluted in a water bath for 20 minutes. 10% serum was then added for 30 minutes at 37°C. A second round of incubation with primary and secondary antibodies was repeated under the same conditions, followed by another 10-minute tyramine salt application at room temperature, shielded from light. Sections were counterstained with DAPI, sealed with a fluorescent sealer, and stored at 4°C in a light-protected environment. Non-specific IgG-stained sections served as negative controls. Imaging was performed using an Olympus microscope.

#### Histological analysis

Changes in vascular collagen and elastin content, as well as aortic structure, were histologically evaluated. Mouse aortas were fixed in 4% paraformaldehyde, embedded in paraffin, and sectioned at 5μm thickness. Masson trichrome and Verhoeff’s van Gieson staining were performed using kits from BIOSSCI Biotech (Hubei, China) to observe the overall morphology and elastin within the aortic wall, respectively. The collagen and elastin content were quantified by scaling the sampled area to the total tissue area.

#### Oil red O staining

Section frozen liver samples to a thickness of 10 μm, and rinse the fixed sections or cells in 60% isopropanol. Prepare the staining solution by mixing oil red O stock solution with distilled water, allowing it to sit for 10 minutes. Stain the sections for 5-10 minutes, then remove excess dye with 60% isopropanol and wash with distilled water. Subsequently, apply hematoxylin to stain the nuclei. Lipid droplets will appear red, and analysis of stain-positive regions was performed using Image J.

#### Transmission electron microscopy (TEM)

The extent of mitochondrial damage and autophagy levels in macrophages were evaluated using TEM. Vascular tissue was fixed in electron microscopy fixative and stored at 4°C. It was subsequently fixed in 0.1M phosphate buffer with 1% osmic acid for 2 hours, dehydrated with alcohol and acetone, and embedded in 812 Embedding Medium, which was polymerized at 60°C for 48 hours after an overnight bake at 37°C. The polymerized tissue was sectioned into 80 nm slices, mounted on a copper grid, stained, and examined under a transmission electron microscope (HITACHI; HT7700).

#### Cell culture

Bone marrow-derived macrophages (BMDMs) were harvested from the bone marrow of 8-week-old male C57BL/6 mice and cultured in 5 mmol/L glucose DMEM (Gibco, USA) supplemented with 10% FBS, 1% penicillin-streptomycin, and 20 ng/mL M-CSF (PeproTech, USA) for 1 week to induce maturation. After 1 week of differentiation, BMDMs were cultured for 72 hours under varying glucose concentrations (5 mmol/L glucose DMEM, with additional glucose to achieve a final concentration of 25 mmol/L). Senescence was confirmed by tests for senescence-associated markers (p16, p21, p53) and Senescence-Associated Beta-Galactosidase (SA-β-gal) activity.^14^^,^^79^

VSMCs isolation and culture: The aorta of 6-week-old mice were isolated, cleared of adipose tissue, and cut into small tissue blocks. These blocks were placed in a Petri dish and allowed to dry slightly for 20 minutes in a cell culture incubator to ensure adhesion to the bottom of the cell flasks. DMEM/F12 medium with 20% FBS was then added, and the cells were cultured statically for 6 days. Primary VSMCs that began to proliferate were further cultured in DMEM/F12 medium with 10% FBS and 1% penicillin/streptomycin for subsequent experiments.^29^

Co-culture: Macrophages were seeded in the upper layer of a Transwell coculture system with a 0.4 μm pore size six-well plate. Macrophage senescence was induced by HG intervention when cell density reached 70%-80%. Vascular smooth muscle cells, seeded in plates taken in the lower layer of the coculture system, had a cell count of about 0.5×10^6^ cells per well. The macrophage-seeded Transwell chambers were gently inserted into the upper layer of the vascular smooth muscle cell-seeded culture plate using sterile forceps. Due to the design of the coculture system, the Transwell chambers were suspended above the culture plate, allowing free exchange of soluble factors between macrophages and vascular smooth muscle cells.

#### SA-β-gal staining

SA-β-Gal staining was performed using the SA-β-gal staining kit (Beyotime, China; C0602). After washing the cells three times with PBS, they were fixed for 15 minutes at 37°C and incubated overnight with freshly prepared SA-β-gal staining solution. For histologic evaluation, vascular samples were fixed for 20 minutes and incubated overnight in SA-β-gal staining solution at 37°C for 18 hours. Cell nuclei were restained with Hochest 33342 for 10 minutes before imaging.

#### Western blotting

Fresh vascular tissue or cells were lysed, and total protein was extracted and quantified. Approximately 30 μg of total protein was loaded onto an SDS/PAGE gel. The separated proteins were then transferred to a polyvinylidene difluoride (PVDF) membrane, which was blocked in 5% skim milk containing PBS/0.1% Tween for 1 hour. The primary antibody is then incubated overnight at 4°C. The next day, the membrane was rinsed with PBS/0.1% Tween and incubated with secondary antibody for 1 hour. Antibody labeling was visualized using an electrochemiluminescence system (Bio-Rad, Hercules, CA, USA). All primary antibodies used in this research are listed in key resources table.

#### RNA isolation and qPCR

BMDMs were harvested, and total RNA was extracted using TRIzol reagent (15596026; Invitrogen, CA, USA) following the manufacturer’s protocol. Subsequently, cDNA was then synthesized using the RevertAid First Strand cDNA synthesis kit (K1622; Thermo Fisher Scientific), as per the manufacturer’s directions. Quantitative reverse transcription PCR (qPCR) was conducted in duplicate using SYBR Green Supermix (1725124; Bio-Rad, CA, USA). The relative mRNA levels of each gene were quantified using the 2−ΔΔCT method, and primer sequences are listed in Table 1.

#### Isolation of PBMC

The PBMC isolation method was adapted from prior studies.^80^ EDTA-anticoagulated blood from C57BL/6 mice was mixed with an equal volume of RPMI 1640 medium. PBMCs were separated using a mononuclear cell isolation solution (DAKEWE, Cat#：DKW33-R0100) by density gradient centrifugation. After centrifugation at 800g for 30 minutes, the mononuclear cell layer was collected and washed three times.

#### ROS detection

ROS production was measured using a ROS Assay Kit (Beyotime, China). Cells were treated with 2′,7′-dichlorodihydrofluorescein diacetate (DCFH-DA) (10 μM) in fresh medium and incubated in the dark for 20 minutes. Observations were made using a fluorescence microscope (IX73, Olympus, Tokyo, Japan) and an NL-3000 Northern Light flow cytometer (Cytek Biosciences, Fremont, CA, USA).

#### Mitochondrial membrane potential measurement

The enhanced mitochondrial membrane potential assay kit with JC-1 (C2003S, Beyotime) was used to detect mitochondrial membrane potential. After cell staining, fluorescence images were captured using a laser confocal microscope (Carl Zeiss LSM710; Germany).

#### Cytoplasmic mtDNA detection

Wash BMDMs with HBSS and incubate with Mito-Tracker Red (C1034, Beyotime, China) for 0.5 hours. After a second HBSS again, incubate with Picogreen (12641ES01, Yeasen, China) for 0.5 hours. Then, use a laser confocal microscope immediately to observe the distribution of mitochondrial-like nuclei in the mitochondrial structure of BMDMs.

#### ELISA analysis

Following the methodology outlined in the literature,^81^ approximately 1 × 10^6^ BMDM cells per well in 6-well plates were digested and centrifuged. The cells underwent lysis through repeated freeze-thaw cycles. After centrifugation at 500 × g for 10 minutes at 2-8°C, the supernatant was collected for analysis. We determined the cGAMP concentration using a cGAMP ELISA Kit (501700, Cayman Chemical). Additionally, IL-6 and IL-1β levels in the supernatant were measured following the supplier’s recommended protocol using ELISA kits (JLW20268 and JLW18442, Jianglai, China). For the assay, 50 μL of each sample was added to designated wells and incubated at 37°C for 30 minutes. This was followed by the addition of 100 μL of enzyme conjugate to each well and a further incubation at 37°C for 60 minutes. After four washes, 50 μL of Substrate A and 50 μL of Substrate B were added to each well, and the wells were incubated at 37°C for 15 minutes. Finally, 50 μL of stop solution was added, and the optical density was measured at 450 nm.

#### Co-immunoprecipitation

Co-immunoprecipitation was conducted according to the rProtein A/G Magnetic IP/Co-IP Kit instructions (ACE, China; #BK0004-02). After removing the culture medium, cells were washed twice with precooled PBS. Precooled 1× Lysis/Wash Buffer (Enhanced) was added, and the mixture was incubated for 20 minutes on ice. The lysed sample was then transferred to a centrifuge tube and centrifuged at approximately 13,000 × g for 10 minutes to remove cell debris. To a 1.5 mL centrifuge tube, 20 μL (0.2 mg) of rProtein A/G MagPoly Beads were added, followed by 180 μL of 1× Lysis/Wash Buffer (Enhanced), and washed with magnetic beads. Magnetic beads were co-incubated with 5 ug of STING antibody at 4°C for 16 hours. Subsequently, 700 ug of cell lysate was added to the tube and co-incubate with the magnetic beads and antibody at 4°C for 16 hours. Use a magnetic separator to separate the magnetic beads and retain the sample buffer containing the target antigen.

#### Autophagy monitoring

To monitor autophagic flux, an adenovirus driving mCherry-GFP-LC3B expression was transfected using AdPlus-mCherry-GFP-LC3B (Beyotime, China; C3012). Accumulated LC3 spots were observed by laser confocal microscopy (CarlZeiss LSM710; Germany).

### Quantification and statistical analysis

Results were expressed as mean ± standard error of the mean (S.E.M.), An unpaired t-test was employed to compare a variable between two groups. One-way ANOVA was utilized to assess variability among multiple groups, followed by Bonferroni’s multiple comparison test. A *p* value of <0.05 was considered statistically significant.

### Additional resources

#### Institutional review board statement

All experiments involving animals were conducted according to the ethical policies and procedures approved by the Ethics Committee of the Second Xiangya Hospital of Central South University, Changsha, China (Approval no. 2022400) and in line with the Guide for the Care and Use of Laboratory Animals, NIH publication, 8th edition, 2011.
