# Supplementary material for: Aberrant STING activation promotes macrophage senescence by suppressing autophagy in vascular aging from diabetes
Source: iScience. 2024 Dec 13;28(1):111594. doi: 10.1016/j.isci.2024.111594 (PMC11742833; doi:10.1016/j.isci.2024.111594)
Supplement: Document S1. Figures S1–S5 [file mmc1.pdf]

iScience, Volume *28*

Supplemental information

Aberrant STING activation promotes macrophage senescence by suppressing autophagy in vascular aging from diabetes

Huiqing Ding, Quan Zhang, Rukai Yang, Liyao Fu, Hejun Jiang, Qingyi Zhu, and Shi Tai

A

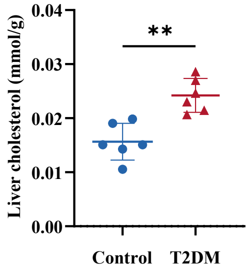

Figure S1. Accelerated vascular aging in T2DM. (A) Cholesterol concentrations were quantified in liver homogenates from control and T2DM mice via colorimetric assays (n=6).

A

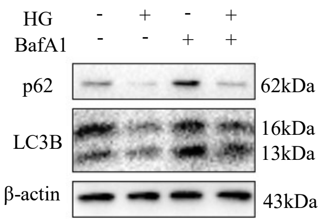

Figure S2. Senescent macrophages accelerate VSMCs senescence. (A) Levels of p62 and LC3 in macrophages were measured by WB, both under conditions of HG and following Bafilomycin A1 treatment.

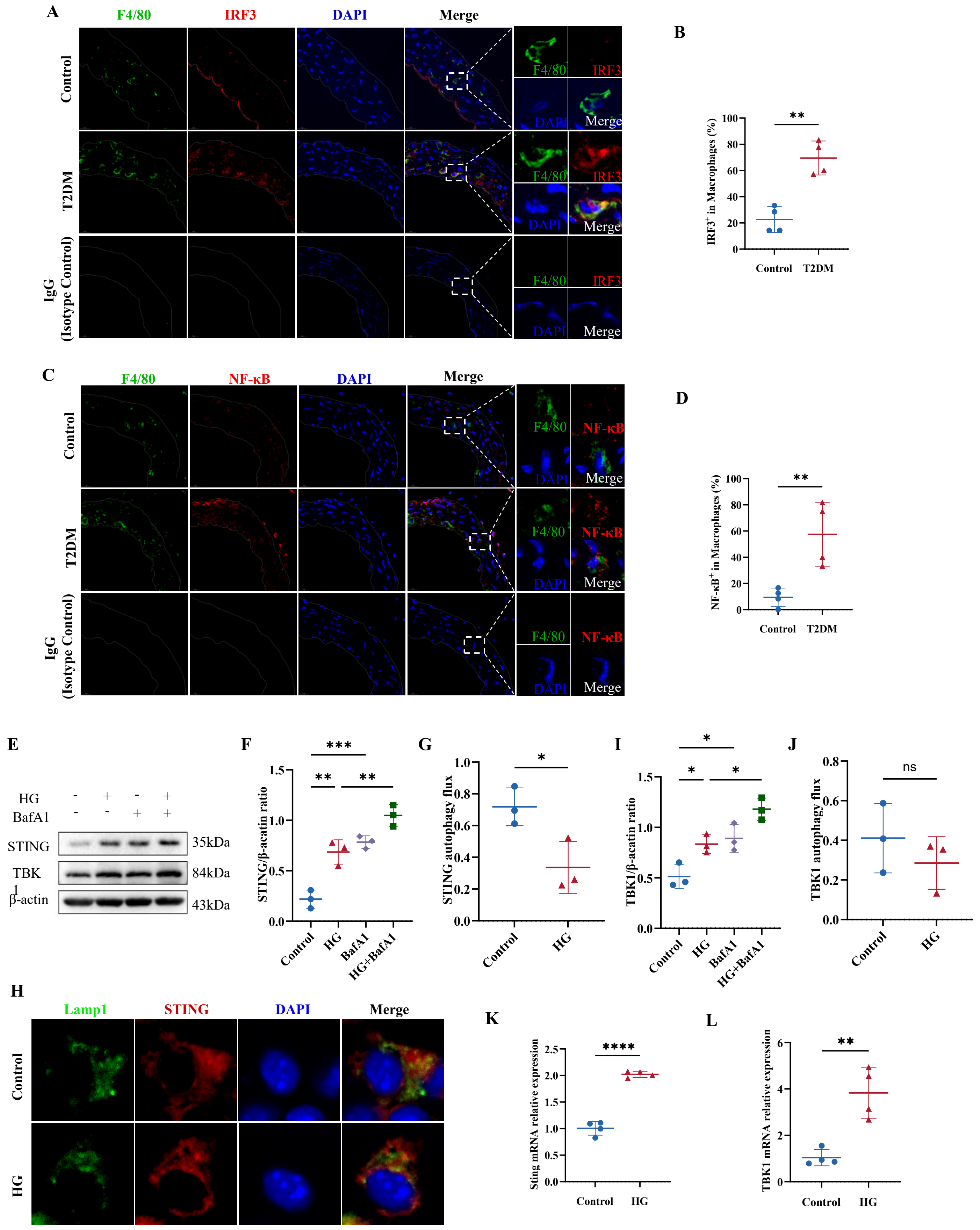

Figure S3. STING mediates macrophage senescence in diabetes mellitus. (A-D) IF staining of F4/80, IRF3, and NF-κB in the aortas of diabetic mice (n=4). Scale bar: 20μm. **(E-G)** Protein concentrations of STING and TBK1 were evaluated under high glucose conditions using WB, incorporating the lysosomal acidification inhibitor Bafilomycin A1 (n=3). **(H)** IF staining of LAMP1 and STING to evaluate co-localization. Scale bar: 1μm. **(I-J)** WB analysis of TBK1 to assess lysosomal degradation under HG conditions (n=3). **(K-L)** Transcription levels of STING and TBK1 were quantitatively analyzed using qRT-PCR (n=4).

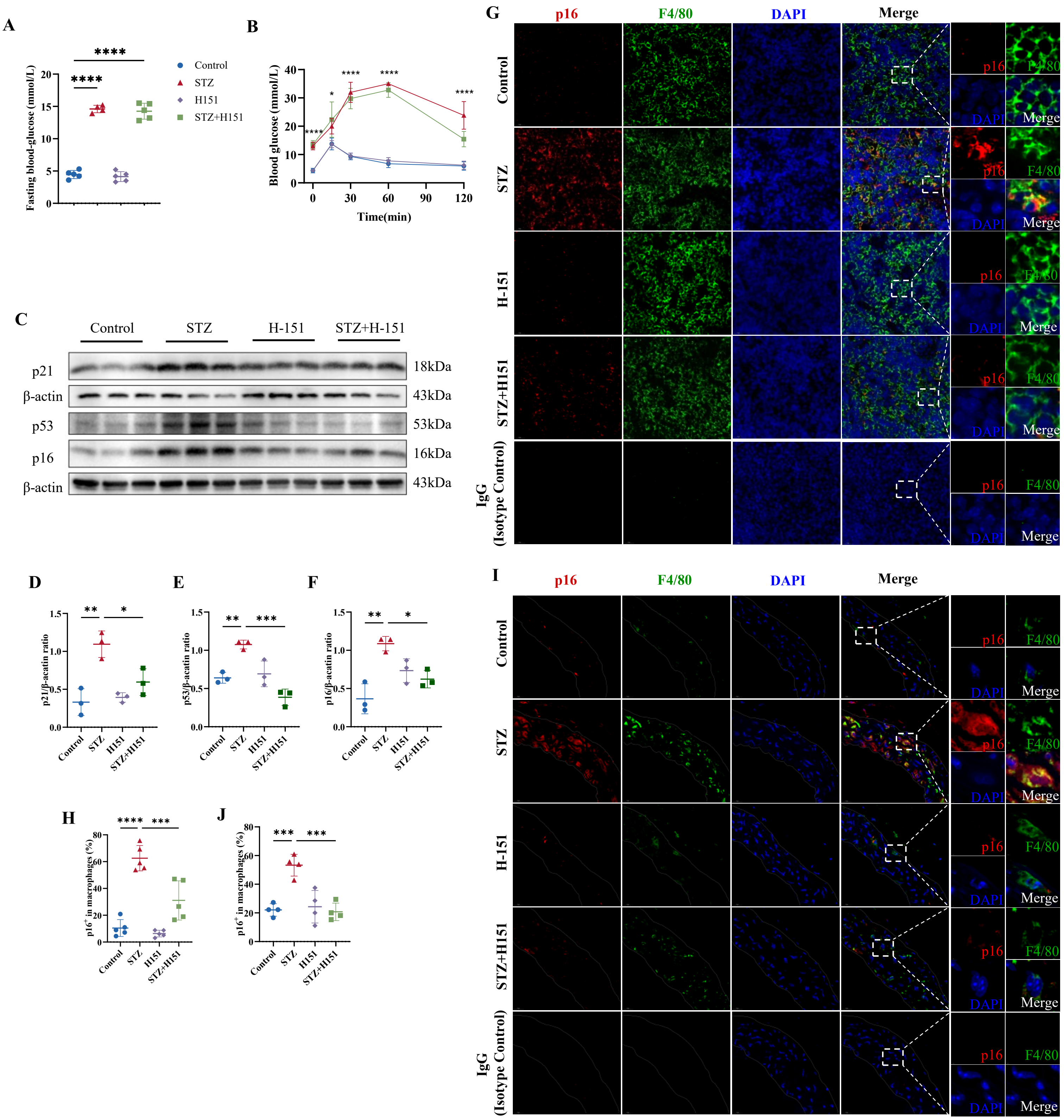

Figure S4. STING promotes senescence of macrophages exposed to high glucose. (A) Measurement of fasting blood glucose levels in mice to confirm the establishment of the diabetes model (n=4-5). **(B)** An IPGTT was performed to evaluate glucose responses in the mice (n=4). **(C-F)** Analysis of protein levels of p53, p21, and p16 in PBMCs by WB (n=3). **(G-H)** IF staining of spleens from four groups of mice to detect p16 and F4/80, assessing the expression level of p16 in macrophages (n=4). **(I-J)** IF staining in the aortas of four groups of mice to detect p16 and F4/80, assessing the expression level of p16 in macrophages (n=4).

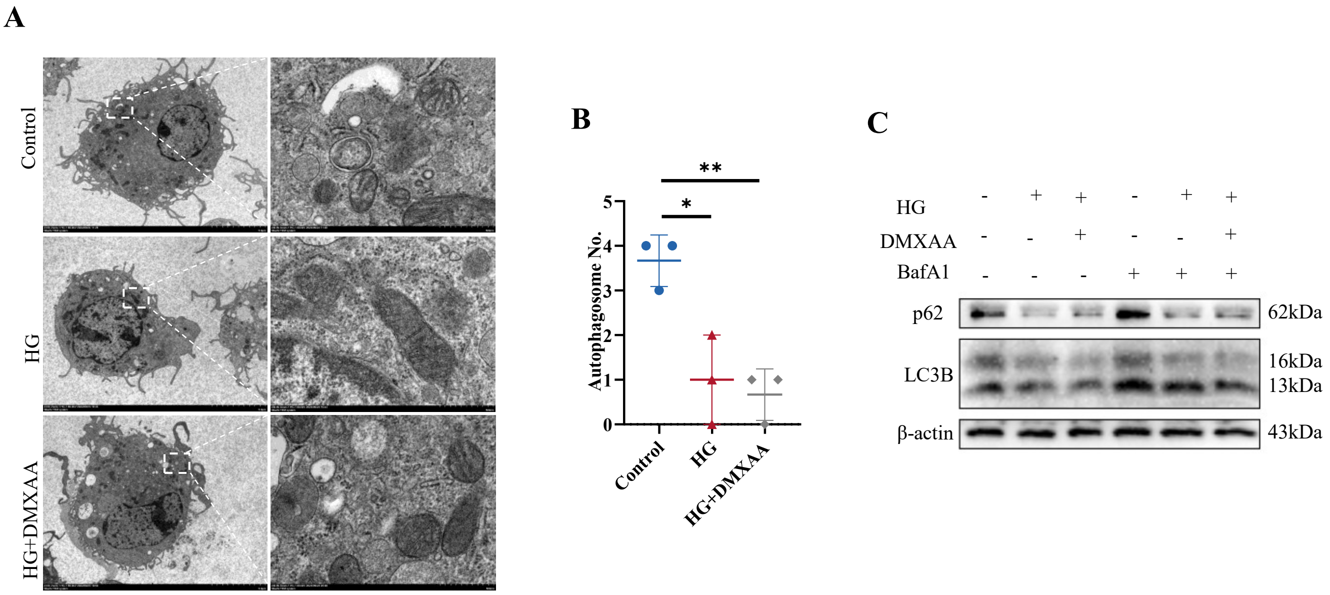

Figure S5. Autophagy flux is inhibited by prolonged STING activation. (A-B) The presence of autophagic vesicles in macrophages treated with DMXAA under HG conditions was observed using TEM. **(C)** WB analysis was performed to assess the levels of autophagy substrates P62 and LC3B.
